# Intraarticular fibroma of tendon sheath

**DOI:** 10.4103/0019-5413.80049

**Published:** 2011

**Authors:** Michael J Griesser, Paul E Wakely, Joel Mayerson

**Affiliations:** Department of Orthopaedics, The Ohio State University, Columbus, OH, USA; 1 Department of Pathology, The Ohio State University, Columbus, OH, USA

**Keywords:** Femoral condyle, intraarticular fibroma, tendon sheath, fibroma

## Abstract

A 17-year-old male presented to us following a hyperflexion injury to his right knee sustained while playing soccer. Immediately after the traumatic event, he developed a large, tense knee effusion. Physical examination revealed limited range of motion. MRI revealed a lobulated mass in the posteromedial aspect of the knee joint. The mass was excised and sections submitted to pathology. A pathologic, microscopic, and immunohistochemical characteristics revealed the final diagnosis of fibroma of tendon sheath in the knee. At 12 months followup, the patient reported no subjective symptoms, such as pain or limitation of athletic activities and has full range of motion. Additionally, he has demonstrated no signs of recurrence. We report a case of fibroma of the tendon sheath originating from the synovial membrane of the joint capsule of the knee.

## INTRODUCTION

Fibroma of tendon sheath (FTS), first described by Geschickter and Copeland in 1949,^1^ is a slow-growing fibrous nodule that frequently adjoins a tendon sheath, and has a predilection for occurring on the fingers and hands. Often affecting middle-aged patients, the onset is usually marked by noticing a small mass or swelling. FTS rarely originates from the synovial membrane of a joint capsule, similar to nodular fasciitis

To our knowledge, only 11 cases of FTS originating from the synovial membrane of a joint capsule have been reported in the English literature, including 6 from the knee, 1 from the radioulnar joint, 1 from the scapholunate joint, 1 in the shoulder joint, 1 in the ankle joint, and 1 in the temporomandibular joint.^2^–^13^ We report here such a case of fibroma of the tendon sheath originating from the synovial membrane of the joint capsule of the knee, and only the second case that demonstrated erosion into bone.

## CASE REPORT

**Figure 1:**
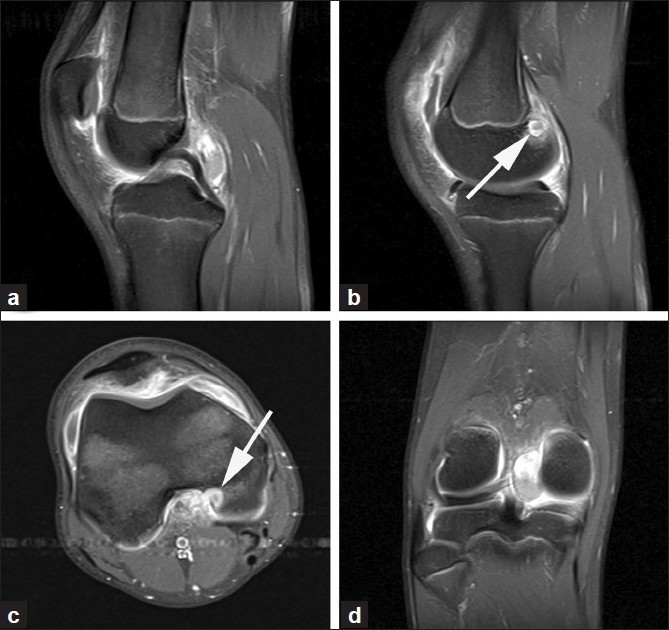
MRI sagittal T1W images showing the lesion is posterior to the posterior cruciate ligament (a). Another view (b) indicates the lesion (arrow) eroding into the femoral condyle. Axial image (c) shows the lesion (arrow) eroding into the posterior medial femoral condyle. Coronal view (d) shows the lesion as it lies laterally to the medial femoral condyle.

A 17-year-old-male presented to us following a hyperflexion injury to his right knee sustained while playing soccer. Immediately after the trauma he developed a large, tense knee effusion and was seen at another institution. The knee was aspirated, and a significant amount of blood was removed. Based on the mechanism of injury as well as the bloody effusion, ligamentous tears were suspected, and a magnetic resonance imaging (MRI) was done. He was referred to our institution 2–3 weeks later. The patient reported having significant pain and limitations in his right knee associated with squatting motion or hyperflexion activity. Examination revealed limited range of motion of the knee joint from 0° to 110° of flexion of the right knee (contralateral knee = 0°–140°). There was mild tenderness to palpation posterior to the right knee, with no evidence of effusion or a palpable mass after 3–4 weeks postinjury. The MRI revealed a lobulated mass in the posteromedial aspect of the knee joint, measuring 3.3 × 1.7 × 1.9 cm at its greatest dimension. The lesion was adjacent to the posterior cruciate ligament (PCL) and appeared densely adhered to both the PCL and the posterior joint capsule. The posterior medial femoral condyle had cortical involvement measuring 1.1 × 0.9 cm and was associated with a moderate knee joint effusion and associated prominent synovial enhancement and thickening. There was some T1 hyperintense signal with prominent enhancement of the more superior portion of the lesion [Figure 1]. The lesion was isointense on T1-weighted imaging and heterogeneously hyperintense on T2-weighted imaging.

**Figure 2:**
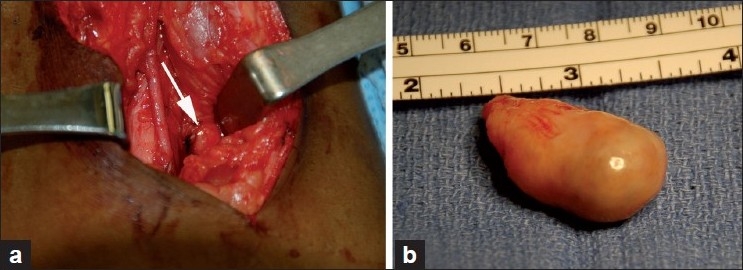
Intraoperative photographs showing a posterior approach to the knee (a) was used to reveal the lesion (arrow). The mass (b) was approximately 3.5 × 2 cm in greatest dimensions

At this point, the preoperative differential diagnosis included a nodular form of pigmented villonodular synovitis (PVNS), ganglion, and synovial chondromatosis. The patient subsequently underwent uneventful excision of the mass using a posterior approach to the knee [Figure 2]. Intraoperatively, the entire lesion was excised and was 3.5 × 2 cm in its greatest dimension. The mass was totally intrarticular and was densely adhered to the synovial membrane of the posterior portion of the joint capsule, the PCL, and the posterior medial femoral condyle. The lesion had some bony penetration as well. As the lesion was very difficult to visualize in its entirety, it was removed sharply from the bone so as not to prohibit a return for greater margins if the frozen section revealed a need to do so. The frozen section was diagnosed as benign intraarticular nodular fasciitis. There were no postoperative difficulties or need for marginal revision.

**Figure 3:**
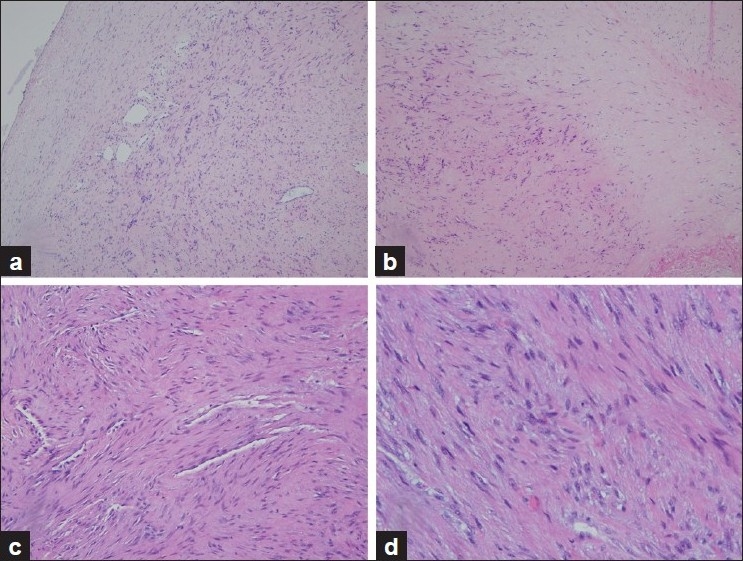
(a) The inked edge of the specimen shows smooth peripheral circumscription (H and E stain, ×20). (b) An abrupt transition from a cellular focus to a hypocellular fibrocollagenous zone is seen (H and E stain, ×20). (c) Bland spindle cells surround parallel slit-like vessels (H and E stain, ×20). (d) A cellular zone shows only a moderate amount of stromal collagen (H and E stain, ×40)

The pathologic specimen grossly consisted of a 3.3 × 1.6 × 1.6 cm tan – white, rubbery mass that weighed 4.5 g. The cut surface was tan–white and rubbery with approximately 15% comprising foci of hemorrhage. The foci of hemorrhage were sampled and showed focal recent hemorrhage and cystic degeneration. Microscopically, a proliferation of uniformly bland, but plump, fibroblastic/myofibroblastic cells were arranged in short intersecting bundles. Markedly elongated nuclei had smooth contours and an open chromatin pattern with occasional distinct nucleoli. The stroma was variably collagenous, harboring microscopic foci of myxoid change and an absence of necrosis, giant cells, hemosiderin deposits, foamy histiocytes, and extravasated red cells. Slit-like vascular channels were particularly noticeable at the periphery of the nodule on one side. Mitoses were rare. Immunohistology demonstrated diffuse positive staining with vimentin and smooth muscle actin with weaker staining for muscle-specific actin. Staining was negative with S-100, CD34, cytokeratin cocktail, and beta-catenin. These pathologic, microscopic, and immunohistochemical characterisitics were suggestive of FTS in the knee [Figure 3].

At 12 months followup, the patient reported no subjective symptoms, such as pain or limitation of athletic activities. Extension of the knee joint was 0° and flexion was 140°, showing no limitation of range of motion. There was no swelling, tenderness to palpation, or signs of recurrence.

## DISCUSSION

FTS is a slow-growing fibrous nodule typically attached to tendon sheath, of which approximately 80% occur in the fingers and hand.^1^,^8^,^14^,^15^ Rarely this lesion has been reported to arise from the synovial membrane of a joint capsule as in our case.^2^

In 1979, Chung and Enzinger^14^ published a report on 138 cases of FTS, which to this day remains the largest series on FTS, and the basis of much of our understanding of these lesions. FTS typically develops in young males in the third to fourth decade, with male to female ratio of 3:1.^14^–^16^ However, most of these cases occur around the fingers, hand, and wrist, and very rarely are reported to be elsewhere anatomically.^14^,^16^,^17^ Additionally, only scattered case reports exist demonstrating FTS of the knee, particularly in an intraarticular location. In all, 26 cases of FTS around the knee have been reported.^8^ However, a detailed report regarding location is lacking in most of these cases. Additionally, only 6 reports have adequately described intraarticular locations for these lesions, with 2 occurring adjacent to the PCL, 1 in the suprapatellar pouch, and 3 in the posterior joint capsule.^2^–^5^,^7^,^18^–^21^ The lesion in our case was densely adhered to the posterior joint capsule, the PCL, and showed bony erosion into the medial femoral condyle. This case is the third originating from the PCL and the fourth emanating from the posterior joint capsule of the knee.

The typical MRI findings in FTS have been described in a case series by Fox *et al*.^22^ This included low intensity on T1-weigthed images in 5 cases, low intensity and isointensity on T2-weighted images in 3 cases, and a slightly high intensity on T2-weighted images in 2 cases. In our patient, MRI revealed some T1 hyperintense signal in the superior portion of the lesion, with T1 isointensity elsewhere and heterogenous hyperintensity on T2-weighted imaging. However, the MRI findings of FTS are very similar to giant cell tumor of tendon sheath (GCTTS) and PVNS and cannot be relied upon for definitive diagnosis.

The only accurate way to diagnose intraarticular nodular FTS is by microscopic examination.^1^,^14^ The principal differential diagnoses include GCTTS, nodular fasciitis, and PVNS. This case was originally mistaken for nodular fasciitis. Several pathologic features typical of that entity were missing, including a lack of extravasated red cells, only minor foci of myxoid change, and an absence of a disordered “tissue culture” appearance to the proliferating spindle cells. Instead, the nodular pattern and the obvious presence of many slit-like small vascular channels is typical of FTS.^23^ Immunohistochemical staining does not distinguish between FTS and nodular fasciitis as both will demonstrate positive staining for smooth muscle actin, muscle-specific actin, and vimentin. GCTTS and PVNS are readily excluded pathologically, based on the absence of giant cells, xanthomatous histiocytes, and hemosiderin deposition. Finally, as more reports of FTS appear in the literature, it would seem prudent to add this condition to the list of differential diagnosis that have historically been considered for a painful, range of motion-limiting, intraarticular mass lesions. Bony penetration or cortical involvement can be seen in GCTTS, PVNS, synovial chondromatosis, calcific tendinitis, and periosteal chondroma, but is rarely reported in FTS or nodular fasciitis.

Entities such as FTS and GCTTS can be treated with marginal excision, alerting the patient and the family that recurrence is possible. Nodular fasciitis, however, can be diagnosed and treated by excisional biopsy, counseling the patient that there may be spontaneous regression of the lesion, even if it is incompletely excised. Symptomatic PVNS is commonly treated with either arthroscopic or open total synovectomy or possibly total joint replacement. Thus, differentiation helps determine treatment as well as how to counsel patients and their families appropriately.

In summary, this case illustrates the importance of an inclusive differential when examining intraarticular knee lesions, one which must include FTS.
